# Mr. Franks, on Variolous Contagion

**Published:** 1801-01

**Authors:** 


### *To the Editors of the Medical and Phyſical Journal.*

Gentlemen,

IN a former communication I made ſome obſervations on the Rival Inoculations, and contended that the Vaccine had not ſo great a degree of ſuperiority over the Variolous, as on firſt reflections might be imagined; for that facts might be enumerated, merated, to ſhew that inoculated Small-pox is very rarely contagious.

With your permiſſion I will now proſecute the enquiry according to promiſe. But, before I proceed to the facts which I intend to adduce, permit me to make a few preliminary remarks.

A part of the ſuperior merit of the Vaccine Inoculation, it is ſaid, conſiſts — in the diſeaſe induced not being infectious.

That the Vaccine Diſeaſe ſhould not infect the human ſpecies unleſs by contact, were I an advocate for the operations of contagions, I would account for; not becauſe it is unattended with ſuppurating puſtules, but from its being a diſeaſe incident to another animal.

That one inoculation ſhould produce infection, and the other not, from the circumſtance of ſuppurating puſtules merely, is not ſatisfactory, unleſs external ſuppuration be regarded as the *sine qua non* of contagion; which is not the caſe, or we ſhould no longer allow ſome other diſeaſes, as typhus, dyſentery, hooping cough, and the eryſipelatous ſore throat to be contagious.

From the evidence which has been adduced, there cannot exiſt a doubt reſpecting the ſecurity which this analogous diſeaſe affords againſt the contagion of Small-pox. And it may be reaſonably ſuppoſed that miſtakes will every now and then occur, which cannot fail to injure the practice, or the profeſſional reputation of the practitioner. Then again, recent matter is more certain of producing infection than when it is dry; and fluid matter is, and probably always will be, more eaſily obtained by the generality of practitioners from Small-pox than from Cow-pox, by reaſon of the locality of the latter diſeaſe. And trifling as the operation of inoculation is, it is to be preſumed that many parents will diſlike thoſe repetitions ſo often neceſſary to communicate the Vaccine Diſeaſe.

Small-pox and Meaſles, though they are diſeaſes which affect the ſyſtem, differ from other univerſal diſeaſes, inaſmuch as they are independent of *excitement*. And it is this circumſtance which renders their *cauſes* leſs apparent than thoſe which induce ſthenic or aſthenic diſeaſes.

It would ſeem that Small-pox is not affected by impurity of air, for it rages with as great violence in villages as in cities.

Diſeaſes of the ſyſtem are preceded by prediſpoſition.—We know that prediſpoſition to general diſeaſe, is either a ſubtraction from, or an addition made, to that juſt degree of excitement which conſtitutes health. But with prediſpoſition to Small-pox we are unacquainted; it nevertheleſs exiſts, with ſome exceptions, throughout the ſpecies, at one time of life or other; other; and it may, for aught we know to the contrary, mount up to diſeaſe frequently, appearing with little or no aſſiſtance from external aids.

That Small-pox is more under the dominion of prediſpoſition than dependant on the foreign powers, appears to be demonſtrated; for we know that many perſons who are obnoxious to the diſeaſe, are not affected by it when an epidemic conſtitution of the air is preſent, and when expoſed to the operation of the whole force of a ſuppoſed contagion; yet, at ſome other period, when the ſtate of the air is unfavourable to this epidemic, and when they have had no communication with Small-pox patients for a great length of time, ſuch perſons become the ſubjects of it.

Prediſpoſition to Small-pox not being dependant on excitement, but of a nature peculiar to itſelf, can induce no other than a diſeaſe *ſui generis*; whilſt prediſpoſition, founded on a morbid degree of excitement, often terminates in diſeaſe different in form but not in eſſence. Thus we obſerve, that a diminution of excitement exiſting amongſt perſons of the ſame community, on their being expoſed to the application of a ſimilar foreign hurtful power, ſhall give occaſion to typhus, dyſentery, or ſome other diſeaſe of the aſthenic claſs. But I ſee no reaſon that contagion ſhould be reſorted to, to account for thoſe ſpecific diſeaſes originating in a *prediſpoſition* not founded on excitement, more than there is to refer to thoſe unknown undefined *ſomethings* to account for *plague*, *typhus*, or *dyſentery*; diſeaſes which are known to be dependant on the *effect* produced by the exciting powers, namely the excitement. And I am of opinion it makes not for Dr. Maclean's arguments to prove, that the *plague never ariſes from contagion*,* his ſuppoſition that Small-pox and Meaſles are the *effects* of ſpecific contagions from the invariableneſs of the diſeaſes induced, and from the conſideration that man is incident to theſe only once during life.

Whether man be incident to ſome diſeaſes which affect the ſyſtem only once during life, or to others which may affect it more than once, I preſume makes no difference, ſo far as relates to *contagions* as *cauſes*. The circumſtance adds nothing to the evidence that ſuch *powers* have no exiſtence; nor has it the leaſt tendency to prove, that any ſpecific contagion capable of inducing a diſeaſe *ſui generis* through the medium of the atmoſphere really exiſts.

---

* A Diſſertation on the Source of Epidemic and Peſtilential Diſeaſes, by Charles Maclean, M. D.

*Effects* are often afcribed to *caufes* which have no exiftence, or, if really exifting, inadequate often to the production of thofe *effects*; and it may frequently happen that Small-pox, inftead of being attributed to *contagion*, might, with greater propriety, be referred to *a variolous epidemic conftitution of the air*, operating with predifpofition fo as to induce the difeafe.

Again;—In confequence of the mortality occafioned by cafual Small-pox, as is apparent from the London Bills having been confiderably augmented fince the introduction of inoculation; from a feeming coincidence of circumftances, by no means regular, as will appear on enquiry, has this increafe been affigned to a *caufe* fufficiently plaufible, as flattering popular prejudice,—*to the practice of inoculation.* Thus has the practice not had fair play; and by means like thefe, have envy, hatred, and malice been excited, not only between the poor and their rich neighbours, but between perfons refident in the fame houfe.

It will appear, from the extracts which I fhall have occafion to make from a valuable work, that the increafe of mortality from Small-pox, commenced long before the introduction of inoculation; and, that it continued to increafe by a regular progreffion, until, from the prevalence of the practice, a decreafe became obfervable. Although a predifpofition not founded on *excitement*, is of a nature unknown to us, yet are we obliged to admit its exiftence, for the reafons which have been affigned; and by this admiffion we extricate ourfelves from thofe difficulties in which contagions involve us.

Predifpofition to Small-pox, is a ftate of the body not acquired from refidence in any particular climate. Man, wherever placed, is endowed with it: the heat of Africa, and the cold of Ruffia, are alike favourable to it.

The univerfality of Small-pox, and the regularity of its recurrence, are circumftances, I think, unfavourable to the idea of its being propagated by *contagion.*

If a difeafe is dependant on *contagion* for its continuance, if the *contagion* be loft, the difeafe would neceffarily difappear. It may poffibly be obferved, that the Variolous Contagion, from having extended its influence over the earth's whole furface, by taking poffeffion of every *pillow* and *bolfter*, *chair* and *table*, and every thing elfe, moveable and immoveable, cannot be deftroyed either by accident or defign; yet, one fhould have expected, that fome given degree of heat would have rendered it inert.

If Inoculated Small-pox be not infectious, how comes it that matter taken from inoculated patients, conveys the diftemper with equal certainty as if it was taken from the natural Small-pox?

pox? has been aſked. Is it not morally certain, ſay they, that the *effluvia* partake of the ſame infectious quality?

To this moral certainty, I beg leave to reply, that *effluvia*, in ſome other inſtances, we know, will not induce diſeaſe; but if the matter of the effluvia be abſorbed, the diſeaſe is occaſioned.

Surgeons and nurſes may by accident inoculate themſelves with ſyphilis, in places appropriated for the reception of venereal patients; but the air of the hoſpital, which is ſtrongly impregnated with effluvia ariſing from the reſpiration, the ulcers, and the abſceſſes of the diſeaſed, is inadequate to produce ſuch an effect.

Practical anatomiſts reſpire an air loaded with putrid effluvia for many hours together, with impunity; whilſt a ſmall quantity of this fluid matter, if abſorbed, ſometimes gives occaſion to a diſeaſe which has proved fatal.

Facts are ſtubborn things, they make an impreſſion on the mind when arguments fail; that I may not incur the diſpleaſure of any of your readers, I will, without further preface, relate ſuch as will ſerve to vindicate a practice which is threatened with diſuſe; and to which we are under great obligations, from a charge which appears not to be well ſubſtantiated, and for theſe I am indebted to Dr. Watkinſon.*

" Medicus, a very eminent and experienced German phyſician, obſerves, in one of his epiſtles to Dr. Petit, of Paris, that the Variolous Contagion is ſo rarely propagated by the artificial diſeaſe, that, although a prodigious number of people have been inoculated, not more than *ten inſtances*, perhaps, can be reckoned, in which it has communicated the infection, notwithſtanding the pains which have been taken to diſcover that pretended quality of it."

" Miege, a celebrated inoculator, declares, that his own experience has afforded but one example of the contagion being propagated by the inoculated Small-pox, and that, that happened by contact, *per oſculum, ideoque proximum per contactum accidit.*"

" Sulzer, who appears to have had conſiderable practice in this art, aſſures Profeſſor Schroëder, of Gottingen, in a letter which he wrote to him in the year 1765, that he had not ſeen *a ſingle caſe in which the inoculated Small-pox had by contagion given the diſeaſe to another.*"

" A ſimi-

* An Examination of a Charge brought againſt Inoculation, by De Haen, Raſt, Dimſdale, and other writers; by John Watkinſon, M. D. Printed in 1777, for J. Johnſon, London.

" A ſimilar aſſurance is given by Dr. Odier, of Geneva, to the author of the *Journal de Medicine*."*

" Mr. Holwell, who reſided upwards of thirty years in the Eaſt Indies, and whoſe account of the manner of inoculating the Small-pox in that country, ſhews the attention that he paid to this ſubject, informs us, that " notwithſtanding the multitudes that are every year inoculated there in the uſual ſeaſon, it adds no malignity to the diſeaſe taken in the natural way, nor *ſpreads the infection as is commonly imagined in Europe*.†"

But the following fact, atteſted by Dr. Schwenke, a phyſician of diſtinguiſhed reputation in Holland, is ſufficient, I think, to remove every doubt that may remain on this head.

" About the end of the year 1767, and the beginning of the year 1768, two hundred people at leaſt, were inoculated at the Hague, who, without much regard either to themſelves or others, frequented all places of public reſort, notwithſtanding which, no epidemic was produced, nor in the whole year did more than eight perſons die of the Small-pox; and of theſe, three died in the ſpring, one by inoculation, and two by the natural diſeaſe, which they had caught at ſome other place and carried with them to the Hague; the remaining five died towards the end of the year. *Vid. M. W. Schwenke Epiſt. in Cel. Sandifort. Biblioth. Med. Tom.* 6."

[ The remainder of this Communication is obliged to be poſtponed at preſent for want of room. ]

